# Robot assisted treatment in psychiatry - fiction or reality?

**DOI:** 10.1192/j.eurpsy.2023.1813

**Published:** 2023-07-19

**Authors:** E. Z. Reininghaus, A. Häussl, I. Zwigl, S. Guggemos, F. T. Fellendorf, N. Dalkner

**Affiliations:** Department of Psychiatry and Psychotherapeutic Medicine, Medical University of Graz, Graz, Austria

## Abstract

**Introduction:**

The evolution of technologies like artificial intelligence and robotics has already begun to shape the future of health care delivery and will have an undeniable impact on patient experiences over the next decades. In times of shortened human resources, especially in the field of health care settings, we should also consider robots as assistance for existing treatment settings. The use of robotic assisted surgery has already found its way into clinical practice and allows doctors to perform many types of complex procedures with more precision, flexibility and control. Nevertheless, to date, the use of robotics in the field of psychiatry is sparse, at least in European countries.

Socially assistive robots (SARs) are robotic technology platforms with audio, visual, and movement capabilities that are being developed to interact with individuals while also assisting them with their management of their well-being. Robots could support classic psychiatric treatment by training cognition and motivation as well as educating patients.

**Objectives:**

The robot “Pepper” has found its home at the Medical University of Graz, Department of Psychiatry & Psychotherapeutic Medicine in Austria in summer 2022. It is friendly and positive, around 1,30m tall, can make conversations, learn people’s tastes, preferences, and habits to help personalize responses and better address needs. He can also offer games, make music and dance.

**Methods:**

In our ongoing studies we use the robot “Pepper” in the context of psychoeducational settings on different mental diseases, training of cognitive functions as well as motivational aspects in inpatients with psychiatric disorders. It can also react and suggest a break during the sessions if he has the impression that participants are stressed or overstrained with content. We collect personal feedback of the patients and associated employees in the hospital through the ongoing usability study, as well as perform a randomized controlled trial to test effects of cognitive and motivational training aspects in comparison to standardized treatment settings.

**Results:**

It is time to apply new technologies in healthcare, especially in times when the staff is decreasing. Better integrating and expanding on the mental health implications of social robots will complement the ongoing drive in the field of psychology and psychiatry to better assist clients with supportive exercises and education, cognitive training, and an asynchronous care option.

**Conclusions:**

Although the use of SARs in mental health research is not yet widespread, new robots and programming are constantly changing, adapting and expanding. There is an abundance of opportunity for growth, expansion, and exploration to triangulate SARs usability and efficacy as the next step in advancing this field. We should not be afraid of this new and expanding technology but come to use it as soon as possible as a support in psychiatric treatment. Let‘s make fiction become reality!

**Disclosure of Interest:**

None Declared

